# Electrophoretic Deposition of Carbon Nanotubes over TiO_2_ Nanotubes: Evaluation of Surface Properties and Biocompatibility

**DOI:** 10.1155/2014/236521

**Published:** 2014-07-01

**Authors:** Jung Eun Park, Il Song Park, Tae Sung Bae, Min Ho Lee

**Affiliations:** ^1^Department of Dental Biomaterials and Institute of Biodegradable Material, Institute of Oral Bioscience and BK21 Plus Project, School of Dentistry, Chonbuk National University, Jeonju 561-756, Republic of Korea; ^2^Division of Advanced Materials Engineering and Institute of Biodegradable Materials, Chonbuk National University, Jeonju 561-756, Republic of Korea

## Abstract

Titanium (Ti) is often used as an orthopedic and dental implant material due to its better mechanical properties, corrosion resistance, and excellent biocompatibility. Formation of TiO_2_ nanotubes (TiO_2_ NTs) on titanium is an interesting surface modification to achieve controlled drug delivery and to promote cell growth. Carbon nanotubes (CNTs) possess excellent chemical durability and mechanical strength. The use of CNTs in biomedical applications such as scaffolds has received considerable attention in recent years. The present study aims to modify the surface of titanium by anodizing to form TiO_2_ NTs and subsequently deposit CNTs over it by electrophoretic deposition (EPD). Characteristic, biocompatibility, and apatite forming ability of the surface modified samples were evaluated. The results of the study reveal that CNTs coating on TiO_2_ nanotubes help improve the biological activity and this type of surface modification is highly suitable for biomedical applications.

## 1. Introduction

The material surface of a dental implant that is used as an artificial replacement tooth should have optimal biocompatibility, because it is constantly in direct contact with vital tissues. In addition, the implant-tissue interaction should promote bioactivity and physical, chemical, and electrical compatibilities. Titanium and titanium alloys have been widely used as dental materials because they optimally satisfy these requirements [1, 2]. However, these materials are also associated with a drawback of buildup of native oxide film that has a low density and unevenly distributed composition. To address this problem, studies have focused on improving the biocompatibility of alloys by promoting the formation of a dense oxide film by using electrochemical techniques [3–5]. Among the methods developed to date, the formation of a titanium oxide (TiO_2_) nanotube (NT) layer on the titanium surface by anodic oxidation has been gaining attention. This layer is chemically bonded to the titanium base material and exhibits enhanced bonding strength at the alloy-substrate interface [6].

Carbon nanotubes (CNTs) have a unique chemical resistance and mechanical strength, as well as excellent electrical and thermal properties and structural features. For these reasons, CNTs have been used in a wide range of electrochemical and biological applications, such as electrically and thermally conductive composites and biosensors, as well as drug delivery systems, dental implantations, and bone formation. Its application also extends to the field of biomedical engineering for regenerative medicine, such as CNT scaffold materials [7–10]. Previous studies have shown that CNT-coated titanium specimens more efficiently bound cells compared to that by the uncoated titanium specimens [11]. This implies that the strong cell-binding effect of CNTs makes them an excellent material for inducing strong fusion between dental implant materials and periodontal ligaments. Current coating methods for CNTs include plasma spray  [12], aerosol deposition [13], and electrodeposition [14]. In this study, electrodeposition was used because of its advantages of being cost-effective, ability to form a film of uniform thickness, and homogeneous properties irrespective of the surface shape of the implant [15, 16]. We used multiwalled carbon nanotubes (MWCNTs) by using a carboxyl group (COOH) to enhance the dispersibility.

The specimens used in this study were fabricated by forming TiO_2_ NTs on the surface of the titanium alloy, which is the most widely used material for bone replacement, and coating the surface with carboxylated multiwalled carbon nanotubes (MWCNTs-COOH) by electrodeposition. We then investigated the cell proliferation and biocompatibility according to surface modifications.

## 2. Experimental Materials and Methods

### 2.1. TiO_2_ NT Specimen Fabrication

To fabricate specimens for this study, Ti-6Al-4V (Kobe Steel Ltd., Japan) was cut into 20 × 10 × 2 mm portions. All specimens were polished with silicon carbide (SiC) sand paper (numbers 400–1000) to achieve surface homogeneity, followed by ultrasonic cleaning and drying with ethanol and distilled water. To fabricate titanium alloy NTs, the titanium alloy and platinum sheet were connected to the anode and cathode electrodes, respectively, of a DC power supply device (SDP-303D, Daunanotek, Korea). An electrolyte solution was prepared by mixing ethylene glycol (HOCH_2_CH_2_OH; 90 wt%) and ammonium fluoride (NH_4_F; 1 wt%). TiO_2_ NTs were fabricated by applying 20 V for 50 min and then were subjected to heat treatment at 450°C for 2 h to achieve the densification of the TiO_2_ NTs oxide film and structural stabilization.

### 2.2. CNT Preparation

The MWCNTs (CNT Co. Ltd., Korea) used in this study underwent heat treatment at 450°C for 90 min to remove metal catalysts and amorphous carbon and then sonication in a 6 M HCl solution for 2 h, followed by stirring in a round flask and mixing it with a 3 M NaOH solution at 120°C for 12 h. After the stirring, the CNTs were cleaned until the mixture reached a pH level of 7 and then dried at a 55°C vacuum condition until only pure CNTs were left.

To improve dispersibility, the purified CNTs were transferred into a round flask and subjected to sonication in 60% nitrogen solution for 30 min and stirred at 120°C for 8 h.  After the stirring, the CNTs were cleaned again until they reached a pH level of 7 and then they were dried at a 55°C vacuum condition to harvest the MWCNTs-COOH. To measure the dispersibility of CNTs, we separated pure MWCNTs from the MWCNTs-COOH and allowed these to diffuse in distilled water and analyzed their respective diffusivities by using UV-Vis spectrophotometer (HP 8453, Hewlett-Packard, Germany).

### 2.3. CNT Coating

Approximately 5 mg of MWCNTs-COOH was mixed with 100 mL of ethanol and sonicated in an ultrasonic bath for 1 h for dispersal. To induce electrodeposition, the titanium alloy and the platinum sheet were connected to the anode and cathode electrodes, respectively, of a DC power supply. As the electrolyte solution, a suspension solution was prepared by dispersing MWCNTs-COOH in ethanol, and since the MWCNTs-COOH are negatively charged and the titanium alloy was connected to the anode, the MWCNTs were deposited on the titanium alloy; the electrodeposition was performed at 20 V for 1 min.

### 2.4. Surface Analysis

The surface roughness of specimens was tested using a roughness tester (SV-528, Mitutoyo, Japan).

The surface structure was examined using atomic force microscopy (AFM; MultiMode + Bioscope, Digital Instruments, USA) at the speed of approximately 1 Hz and within a range of 5 × 5 *μ*m^2^, and the surface morphology was examined using a field emission scanning electron microscope (FE-SEM) (SUPRA 40VP, Carl Zeiss, Germany).

### 2.5. *In Vitro* Testing

A synthetic body fluid (SBF) solution was prepared by adding 0.285 g/L of calcium chloride dehydrate, 0.09767 g/L of magnesium sulfate, and 0.350 g/L of sodium hydrogen carbonate to Hank's solution (H2387, Sigma Chemical Co., USA), with the pH adjusted to 7.4. Each specimen was completely soaked in SBF for 10 days. After submersion, the precipitation patterns of the bone-like apatite were analyzed with an X-ray diffractometer (X'Pert Powder, PANalytical, The Netherlands). Additionally, osmium coating was performed and the film components and their compositions were measured using an FE-SEM and energy dispersive X-ray spectroscopy (EDX).  All experiments were carried out in triplicate and analyzed with one-way ANOVA (*P* > 0.05).

### 2.6. Cell Culture and Observation

Cytotoxicity assessment was performed on rat preosteoblastic MC3T3-E1 cells procured from the American Type Culture Collection (ATCC, Manassas, VA, USA). The culture broth was prepared by supplementing the culture medium *α*-MEM (Gibco Co., USA) with 10% fetal bovine serum (FBS, Gibco Co., USA) and penicillin. The prepared specimens were UV-sterilized for 24 h, and the cells were divided, with the cell density controlled at 2 × 10^4^ cells/mL and incubated for 24 h at 37°C in a 5% CO_2_ cell culture chamber (3111, Thermo Electron Corporation, USA). The culture broth was then removed and any detached cells and other suspended materials were washed off three times with phosphate buffered saline (PBS) solution. Adherent cells were initially fixed in 2.5% glutaraldehyde for 2 h, followed by a second fixation in 1% osmium tetroxide at 4°C for 2 h. The cells were then dehydrated in an alcohol series of increasing concentrations (50, 60, 70, 80, 90, and 100%). The cell adhesion patterns were then observed using a SEM (Bio-LV-SEM; SN-3000 Hitachi, Hitachi, Japan).

### 2.7. MTT Assay

For the MTT assay, each specimen was incubated for 1 and 5 days by using the same conditions as those for the cell culture, and then the culture medium was removed. The assay containing 3-(4,5-dimethylthiazol-2-yl)-2,5-diphenyltetrazolium bromide (MTT) and *α*-MEM culture medium was divided into 1 mL units, which was left to react in a 5% CO_2_ culture chamber for 4 h. The absorbance was measured at 540 nm using ELISA reader (Molecular devices, EMax, US) after removing the culture medium and inducing color revelation with dimethyl sulfoxide (DMSO). All experiments were carried out in triplicate and analyzed using one-way ANOVA (*P* > 0.05).

## 3. Results

**Figure 1:**
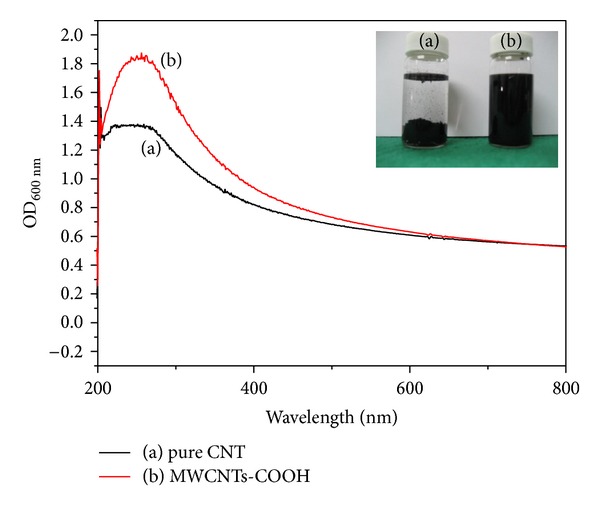
Optical view of dispersion and UV-Vis curve image after dispersion: (a) pure MWCNTs, (b) MWCNTs-COOH.

Prior to CNT deposition on TiO_2_ NTs, MWCNTs dispersibility was assessed after a 1 h sonication of pure MWCNTs and MWCNTs-COOH in distilled water. By visual assessment, pure MWCNTs were found clustered together without being dispersed, thus forming a separate layer from that of distilled water and leaving a clear upper layer (Figure 1(a)), whereas MWCNTs-COOH particles were well dispersed and evenly distributed in distilled water (Figure 1(b)). To quantitatively assess the CNT dispersibility in distilled water, absorbance was measured using UV-Vis (Figure 1). Figure 1 shows that the absorbance at a wavelength of 300 nm was relatively higher in MWCNTs-COOH (b) compared to pure MWCNTs (a).

**Figure 2:**
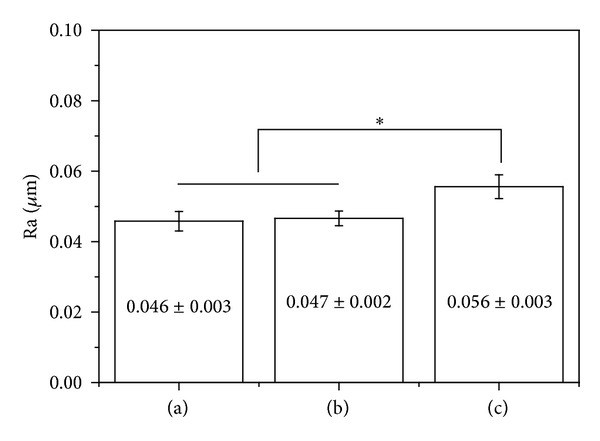
Roughness of (a) Ti-6Al-4V, (b) TiO_2_ NTs, and (c) MWCNTs-TiO_2_ NTs (*P < 0.05).

Figure 2 shows surface roughness according to different surface treatments and the coating of TiO_2_ NTs with MWCNTs-COOH. No significant differences in the surface roughness between Ti-6Al-4V alloy and TiO_2_ NTs were observed (*P* > 0.05), although surface roughness increased with CNT coating (*P* < 0.05).

**Figure 3:**
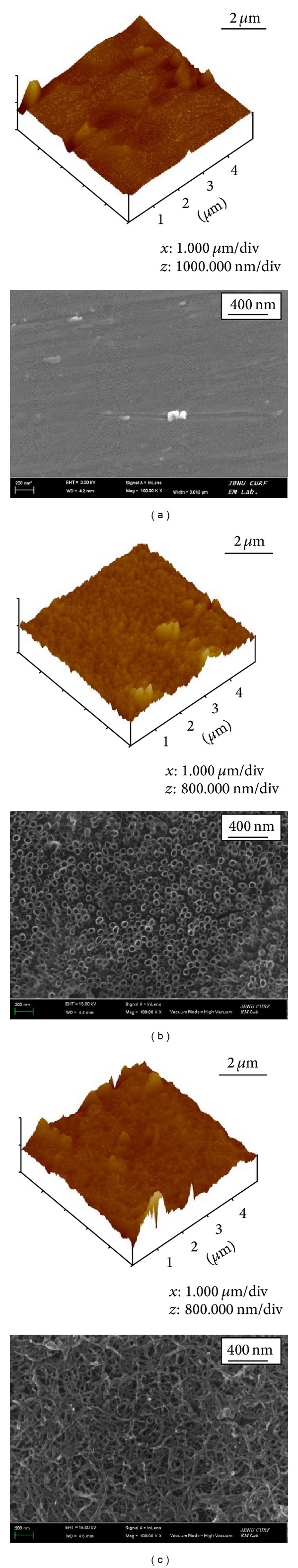
AFM and SEM of Ti specimens surface: (a) Ti-6Al-4V, (b) TiO_2_ NTs, and (c) MWCNTs-TiO_2_ NTs.

Figure 3 shows the AFM and SEM images in which the surface of each specimen was analyzed. Comparison of AFM and SEM images indicated similar surface formation patterns. Moreover, the formed TiO_2_ NTs measured approximately *⌀* 70 nm (Figure 3(b)), whereas the MWCNT-coated surface measured approximately *⌀* 40 nm (Figure 3(c)) on titanium alloy.

**Figure 4:**
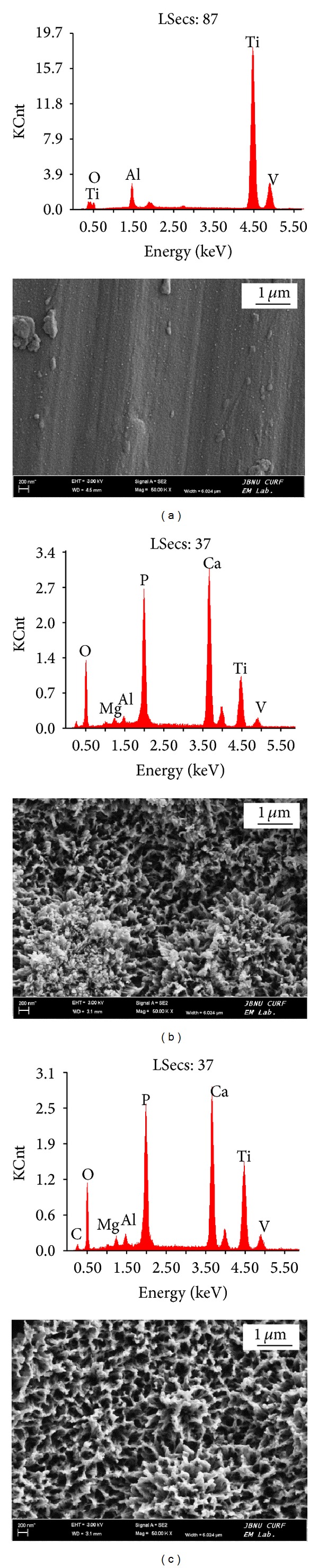
SEM and EDX image of sample immersed in SBF after 10 days: (a) Ti-6Al-4V, (b) TiO_2_ NTs, and (c) MWCNTs-TiO_2_ NTs.

Figure 4 shows the results of analysis of the surface of each specimen with EDX and SEM after immersion in SBF for 10 days. EDX examination did not detect calcium (Ca) and phosphorous (P) in the untreated specimen (a), whereas both were detected on the surfaces of TiO_2_ NT specimen (b) and MWCNTs-TiO_2_ NTs specimen (c).

**Figure 5:**
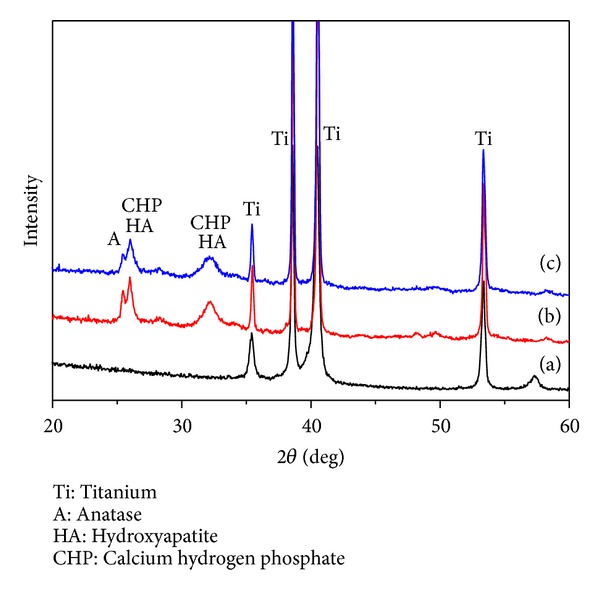
XRD patterns of (a) Ti-6Al-4V, (b) TiO_2_ NT, and (c) MWCNTs-TiO_2_ NT soaked in SBF for 10 days.

Figure 5 shows the results of XRD analysis of each specimen of Ti-6Al-4V alloy (a), TiO_2_ NTs (b), and MWCNTs-TiO_2_ NTs (c) after immersion in SBF for 10 days. In the specimen with TiO_2_ NTs, 2*θ* peaks were found around 31-32° for hydroxyapatite (HA) crystals and at around 25° for anatase (Figures 5(b) and 5(c)).

**Figure 6:**
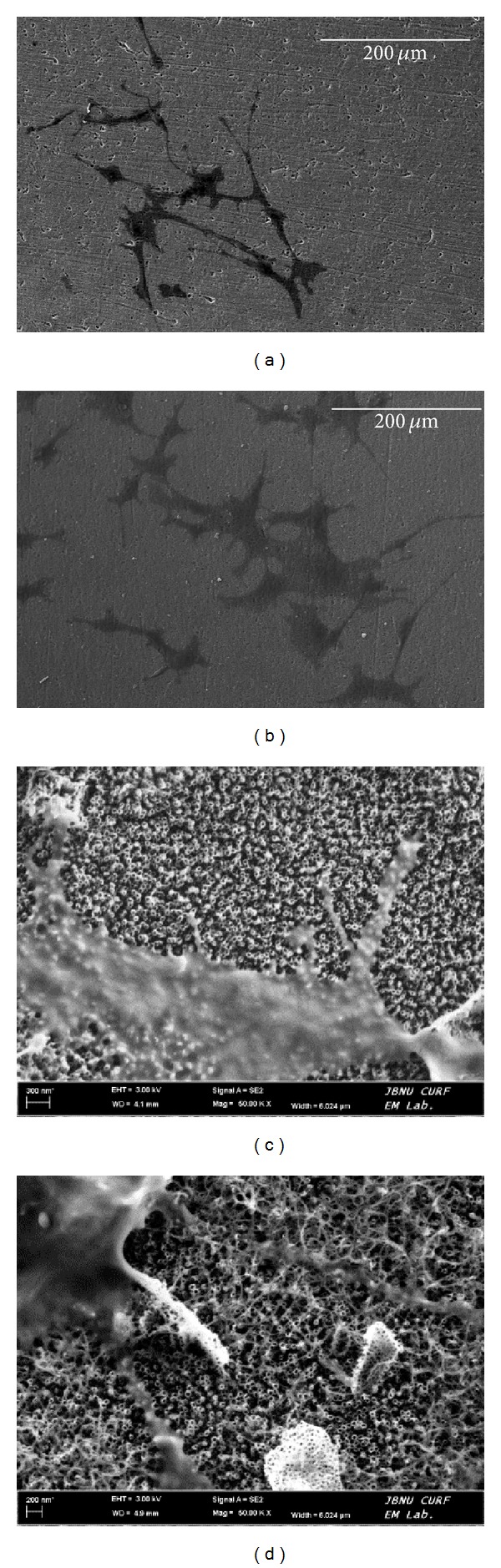
Bio-SEM of MC3T3-E1 cell culture on Ti specimens for 48 h: (a) TiO_2_ NTs, (b) MWCNTs-TiO_2_ NTs; (c) and (d) magnify, respectively, (a) and (b).

Figure 6 shows the cell morphologies in each specimen after a 48 h cell culture. It was confirmed that cells adhered to the surface.  All specimens showed similar patterns with respect to cell morphology, although the cells in the MWCNTs-TiO_2_ NTs were larger and rounder and had more densely developed filopodia (Figure 6).

**Figure 7:**
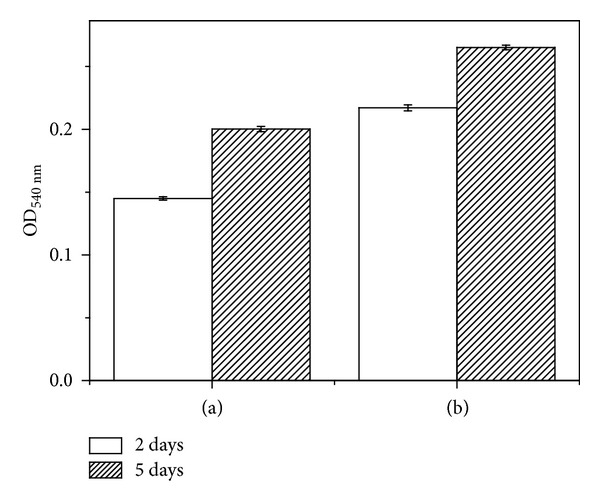
MTT assay of (a) TiO_2_ NTs and (b) MWCNTs-TiO_2_ NTs by MC3T3-E1 cell culture for 2 and 5 days.

Figure 7 shows the results of the MTT assay, which examined cell proliferation after culturing for 2 and 5 days. On incubation day 2, MWCNTs-TiO_2_ NTs specimen showed a large increase in cell proliferation compared to TiO_2_ NTs (*P* < 0.05). Day 5 showed a relatively greater cell proliferation compared to that observed on day 2. In particular, cell survival decreased after the formation of TiO_2_ NTs, but cell viability significantly increased after MWCNT coating on TiO_2_ NTs.

## 4. Discussion

On the basis of their properties of electrical conductivity and chemical/physical, CNTs offer a wide spectrum of application not only for various electrically and thermally conductive composites and chemical sensors but also as nanobiomaterials [11]. Intensive research activities utilizing these superior CNT properties are currently being explored in various fields, but the large specific surface area of CNTs causes NTs to cluster together, often resulting in difficulties in their applications. Despite continuous research on the dispersibility of CNTs, no cure-all solution has been reported to date. Among the common methods of improving the dispersibility of CNTs, several studies have focused on surface functionalization, which involves weakening of the van der Waals force among tubes, thus, preventing their agglomeration through the effects of other materials applied onto the NT surface. One of these methods is the sulfate- and nitrogen-mediated carboxyl application to induce a direct covalent bond between the material and the CNT surface [17–22]. This study similarly conducted an experiment by applying a carboxyl group to the CNT surface by using nitrogen to improve the dispersibility of CTNs. Our results showed that dispersibility increased through the carboxyl group (Figure 1) and, with the CNT surface exhibiting a negative charge, the specimen was connected to the anode for coating.

To improve conditions for osseointegration on the implant surface, studies have explored methods in rendering the surface with micro/nanostructures. A few studies conducted on nanostructured implant surfaces have shown that nanostructures may be more favorable for osseointegration and this may be attributable to its larger specific surface area and not its microstructure [23].  Moreover, given that osseointegration involves a direct contact between the implant surface and cells, changes in surface roughness and surface energy caused by changes in the surface microstructure of the implant surface layers have influenced cell reactivity [24]. We have examined the surface roughness of Ti-6Al-4 alloy, TiO_2_ NTs, and MWCNTs-TiO_2_ NTs and have observed that surface roughness of the MWCNT-coated specimen increased (Figure 2) due to the 40 nm thick MWCNT coating on the surface. Previous studies have shown no consensus on the influence of surface roughness on bone formation, although some reports have shown that rough surfaces, compared to that in smooth surfaces, are generally more favorable to the proliferation and differentiation of osteoblasts and the formation of bone matrix [25, 26]. Additionally, through the adsorption of inorganic ions such as Ca and P, which are present in the body fluid and in bone tissues on titanium implant surfaces, calcium phosphate can be precipitated and crystallized to form apatite phase and simultaneously creates strong bonds with the bone tissue. However, given the fact that it takes several months for osseointegration to occur between the bone tissue and implant, methods for accelerating apatite formation have been investigated. In our study, surface-treated specimens were soaked in SBF solution for 10 days and apatite formation was examined using SEM and EDX. The results showed that Ca and P were precipitated on the titanium surface that has TiO_2_ NT layers (Figure 4). XRD examinations of elementary crystalline structures verified the formation of HA (Figure 5). This may be explained by the initial acceleration of Ca adsorption caused by the OH^−^-supported large surface area of TiO_2_ NT layers, as well as the crystallization of the rapidly deposited Ca and P in the initial phase and subsequent apatite formation. The superior corrosion resistance of titanium is attributable to its extremely high affinity for oxygen and, consequently, strong tendency to build a stable passive film layer on the surface. Nevertheless, an oxide film layer naturally generated in air is not sufficiently thick and dense and is likely to incur pitting corrosion and other degradations. To increase the biocompatibility of titanium, it is essential to form an apatite layer on the implanted metal surface. Researchers have explored methods in creating a micro/nanostructure on the surface layer, along with forming dense TiO_2_ layers to increase its specific surface area, which is considered favorable for osseointegration. With respect to the precipitation of HA, it has been reported that forming micro/nanostructured TiO_2_ layers is more effective than forming dense TiO_2_ oxide film layers [23]. Furthermore, Li et al. [27] reported that a titanium oxide layer forms apatite in SBF and that the Ti-OH group plays an important role in the formation of apatite within the body. According to Webster and Ejiofor [28], the adhesion and proliferation of osteoblasts are promoted when the crystal size constitutes that of nanostructures even when its alloy composition differs, as is the case with titanium and Ti-6Al-4V and Ti-6Al-7Nb alloys.

In order to survive, cells should proliferate after its adsorption and stable establishment on a surface. Preosteoblastic MC3T3-E1 cells develop generally filopodia during their adsorption onto a surface. The examination of the adsorption patterns of MC3T3-E1 osteoblasts on each specimen revealed that the cells on the MWCNTs-TiO_2_ NTs were large and round and that the filopodia more strongly adhered to parts where MWCNTs were present (Figure 6). This is presumably due to the strong cell adsorption that occurred when the filopodia were entangled with MWCNTs. Moreover, cell proliferation decreased on the TiO_2_ NTs surface and increased on the MWCNT-coated specimen, and the extent of increase was greater in specimens incubated for 5 days compared to that observed after 1 day of incubation (Figure 7). This result suggests that the MWCNT-coated surface positively influenced cell proliferation. MacDonald et al. reported that, among the CNT scaffold materials used in regenerative medicine and tissue engineering, single-walled carbons nanotubes (SWCNTs) were entangled strongly with collagen and that such composite materials showed high mechanical strength and cell survival ability [10]. Results of examining cell morphology, migration, and proliferation according to the interactions between cells and cell substrates as well as cell activities exhibited in our survival experiments were also published, stating that these surface modifications promoted bone formation and play an important role in improving osseointegration in titanium-based implant [29].

In this study, an experiment that involved coating MWCNTs on the Ti-6Al-4V alloy surface was conducted and this resulted in a significant increase in surface roughness and cell proliferation. The increase of specific surface area owing to the formation of TiO_2_ NTs also accelerated the formation of HA. Specifically, the specimen whose surface was treated with MWCNTs on its TiO_2_ NT surface showed superior biocompatibility.

## 5. Conclusion

This study developed TiO_2_ NTs on a titanium surface, a material that has been widely used as bone replacement, coated it with carboxylated MWCNT-COOH by means of electrodeposition, and examined cell proliferation and morphology according to surface modifications. Biocompatibility was improved through the combined effect of MWCNT-COOH coating on TiO_2_ NTs, which increased surface roughness, accelerated HA formation, and promoted cell proliferation. On the basis of the above results, we believe that TiO_2_ NTs are effective and excellent bone replacement materials for bone regeneration in a variety of fields such as dentistry and orthopedics.

## References

[1] Garg R, Bhartia P, Bahl IJ, Ittipiboon A (2001).

[2] Brammer KS, Choi C, Frandsen CJ, Oh S, Johnston G, Jin S (2011). Comparative cell behavior on carbon-coated TiO_2_ nanotube surfaces for osteoblasts vs. osteo-progenitor cells. *Acta Biomaterialia*.

[3] Lugscheider E, Weber T, Knepper M, Vizethum F (1991). Production of biocompatible coatings by atmospheric plasma spraying. *Materials Science and Engineering A*.

[4] Fini M, Cigada A, Rondelli G (1999). In vitro and in vivo behaviour of Ca- and P-enriched anodized titanium. *Biomaterials*.

[5] De Andrade MC, Sader MS, Filgueiras MRT, Ogasawara T (2000). Microstructure of ceramic coating on titanium surface as a result of hydrothermal treatment. *Journal of Materials Science: Materials in Medicine*.

[6] Wang B, Lee T, Chang E, Yang C (1993). The shear strength and the failure mode of plasma-sprayed hydroxyapatite coating to bone: the effect of coating thickness. *Journal of Biomedical Materials Research*.

[7] Aoki N, Yokoyama A, Nodasaka Y (2005). Cell culture on a carbon nanotube scaffold. *Journal of Biomedical Nanotechnology*.

[8] Zanello LP, Zhao B, Hu H, Haddon RC (2006). Bone cell proliferation on carbon nanotubes. *Nano Letters*.

[9] Nayak TR, Jian L, Phua LC, Ho HK, Ren Y, Pastorin G (2010). Thin films of functionalized multiwalled carbon nanotubes as suitable scaffold materials for stem cells proliferation and bone formation. *ACS Nano*.

[10] MacDonald RA, Laurenzi BF, Viswanathan G, Ajayan PM, Stegemann JP (2005). Collagen-carbon nanotube composite materials as scaffolds in tissue engineering. *Journal of Biomedical Materials Research A*.

[11] Terada M, Abe S, Akasaka T, Uo M, Kitagawa Y, Watari F (2009). Multiwalled carbon nanotube coating on titanium. *Bio-Medical Materials and Engineering*.

[12] Balani K, Chen Y, Harimkar SP, Dahotre NB, Agarwal A (2007). Tribological behavior of plasma-sprayed carbon nanotube-reinforced hydroxyapatite coating in physiological solution. *Acta Biomaterialia*.

[13] Hahn B, Lee J, Park D (2009). Mechanical and in vitro biological performances of hydroxyapatite-carbon nanotube composite coatings deposited on Ti by aerosol deposition. *Acta Biomaterialia*.

[14] Kaya C (2008). Electrophoretic deposition of carbon nanotube-reinforced hydroxyapatite bioactive layers on Ti-6Al-4V alloys for biomedical applications. *Ceramics International*.

[15] Ishizawa H, Ogino M (1995). Formation and characterization of anodic titanium oxide films containing Ca and P. *Journal of Biomedical Materials Research*.

[16] Ishizawa H, Ogino M (1995). Characterization of thin hydroxyapatite layers formed on anodic titanium oxide films containing Ca and P by hydrothermal treatment. *Journal of Biomedical Materials Research*.

[17] Rastogi R, Kaushal R, Tripathi SK, Sharma AL, Kaur I, Bharadwaj LM (2008). Comparative study of carbon nanotube dispersion using surfactants. *Journal of Colloid and Interface Science*.

[18] Saito T, Matsushige K, Tanaka K (2002). Chemical treatment and modification of multi-walled carbon nanotubes. *Physica B: Condensed Matter*.

[19] Yang C, Kim DY, Lee YH (2005). Formation of densely packed single-walled carbon nanotube assembly. *Chemistry of Materials*.

[20] Marshall MW, Popa-Nita S, Shapter JG (2006). Measurement of functionalised carbon nanotube carboxylic acid groups using a simple chemical process. *Carbon*.

[21] Hirsch A (2002). Functionalization of single-walled carbon nanotubes. *Angewandte Chemie*.

[22] Kumar I, Rana S, Rode CV, Cho JW (2008). Functionalization of single-walled carbon nanotubes with azides derived from amino acids using click chemistry. *Journal of Nanoscience and Nanotechnology*.

[23] Yang B, Uchida M, Kim H-M, Zhang X, Kokubo T (2004). Preparation of bioactive titanium metal via anodic oxidation treatment. *Biomaterials*.

[24] Zhu X, Chen J, Scheideler L, Altebaeumer T, Geis-Gerstorfer J, Kern D (2004). Cellular reactions of osteoblasts to micron-and submicron-scale porous structures of titanium surfaces. *Cells Tissues Organs*.

[25] Ricci J, Spivak J, Blumenthal N, Alexander H, Davies JE (1991). Modulation of bone ingrowth by surface chemistry and roughness. *The Bone-Biomaterials Interface*.

[26] Schwartz Z, Kieswetter K, Dean DD, Boyan BD (1997). Underlying mechanisms at the bone—surface interface during regeneration. *Journal of Periodontal Research*.

[27] Li P, Ohtsuki C, Kokubo T, Nakanishi K, Soga N, de Groot K (1994). The role of hydrated silica, titania, and alumina in inducing apatite on implants. *Journal of Biomedical Materials Research*.

[28] Webster TJ, Ejiofor JU (2004). Increased osteoblast adhesion on nanophase metals: Ti, Ti6Al4V, and CoCrMo. *Biomaterials*.

[29] Ku Y, Chung C-P, Jang J-H (2005). The effect of the surface modification of titanium using a recombinant fragment of fibronectin and vitronectin on cell behavior. *Biomaterials*.

